# Brain-Computer Interface Channel-Selection Strategy Based on Analysis of Event-Related Desynchronization Topography in Stroke Patients

**DOI:** 10.1155/2019/3817124

**Published:** 2019-08-28

**Authors:** Chong Li, Tianyu Jia, Quan Xu, Linhong Ji, Yu Pan

**Affiliations:** ^1^Division of Intelligent and Bio-mimetic Machinery, The State Key Laboratory of Tribology, Tsinghua University, Beijing, China; ^2^Department of Physical Medicine and Rehabilitation, Beijing Tsinghua Changgung Hospital, School of Clinical Medicine, Tsinghua University, Beijing, China

## Abstract

In the last decade, technology-assisted stroke rehabilitation has been the focus of research. Electroencephalogram- (EEG-) based brain-computer interface (BCI) has a great potential for motor rehabilitation in stroke patients since the closed loop between motor intention and the actual movement established by BCI can stimulate the neural pathways of motor control. Due to the deficits in the brain, motor intention expression may shift to other brain regions during and even after neural reorganization. The objective of this paper was to study the event-related desynchronization (ERD) topography during motor attempt tasks of the paretic hand in stroke patients and compare the classification performance using different channel-selection strategies in EEG-based BCI. Fifteen stroke patients were recruited in this study. A cue-based experimental paradigm was applied in the experiment, in which each patient was required to open the palm of the paretic or the unaffected hand. EEG was recorded and analyzed to measure the motor intention and indicate the activated brain regions. Support vector machine (SVM) combined with common spatial pattern (CSP) algorithm was used to calculate the offline classification accuracy between the motor attempt of the paretic hand and the resting state applying different channel-selection strategies. Results showed individualized ERD topography during the motor attempt of the paretic hand due to the deficits caused by stroke. Statistical analysis showed a significant increase in the classification accuracy by analyzing the channels showing ERD than analyzing the channels from the contralateral sensorimotor cortex (SM1). The results indicated that for stroke patients whose affected motor cortex is extensively damaged, the compensated brain regions should be considered for implementing EEG-based BCI for motor rehabilitation as the closed loop between the altered activated brain regions and the paretic hand can be stimulated more accurately using the individualized channel-selection strategy.

## 1. Introduction

According to the estimations, some 16 million people per year experience stroke, from which about two-thirds survive worldwide [1]. Stroke remains the most common cause of disability for adults. Stroke survivors suffer various deficits that generate disability in motor, perceptual, and cognitive functioning [2]. Among these disabilities, motor deficits have a large impact on managing everyday activities [3]. Hemiplegia caused by stroke brings terrible burden on patients and their families, especially with impaired upper extremity, because lack of arm-movement control affects independent daily living.

Effective treatment and therapy for stroke rehabilitation has been the focus of research. Recent studies have demonstrated that electroencephalogram- (EEG-) based brain-computer interface (BCI) has a great potential for motor rehabilitation in stroke patients [4–6], which is hypothesized that closing the loop between cortical activity (imagined or attempted motor intention) and actual movement can restore functional corticospinal and corticomuscular connections [7]. In applications with EEG-based BCI for healthy subjects, motor intention of the unilateral arm or hand can be indicated by the decline in the power of the sensorimotor rhythm, such as event-related desynchronization (ERD) [8–10], in the contralateral sensorimotor cortex (SM1) [11]. Similarly, in stroke rehabilitation with EEG-based BCI, SM1 is chosen to detect ERD [12–15].

The principle behind this approach is to encourage the use of SM1 in the affected hemisphere to induce the reorganization of the original neural circuits for motor control [16]. However, if the affected motor cortex is extensively damaged, then vicariation of function will appear with increased levels of activity in the unaffected hemisphere [17]. Functional magnetic resonance imaging (fMRI) studies have revealed widespread changes in the patterns of brain activation during simple movements of the affected hand after stroke [18, 19], and recruitment in the unaffected hemisphere can also contribute to a good recovery for patients with deficits in the affected motor cortex [20].

Therefore, for stroke patients whose movement of the paretic hand is compensated by other brain region(s), the start location of the loop between motor intention and actual movement is expected to be different not only from that of healthy subjects but also from other patients due to brain reorganization. The objective of this paper, therefore, was to investigate ERD topography of stroke patients in different conditions and compare the classification accuracy of the motor attempt of the paretic hand using EEG data from SM1 or from the channels showing ERD. The closed loop with an individualized start location according to each patient's condition may be able to stimulate the neural circuit of motor control more accurately for stroke patients, especially the ones who develop altered brain regions for compensating affected motor function [16].

In the experiment, EEG was recorded when stroke patients were required to make movement attempts with their paretic hand. The acquired EEG was analyzed to identify the motor intention of the paretic hand.  Section 2 describes the conduct of the experiment, and then, Sections 3 and 4 present and discuss the results of the experiment, respectively.

## 2. Methods

### 2.1. Subjects

The study recruited 15 stroke patients. To be able to respond precisely to the experiment task, the patient should be in a stable condition and capable of communicating with the experimenter. Since the best time for rehabilitation is within 6 months after onset, this experiment selected patients that are less than 6 months after stroke. The inclusion and exclusion criteria were described as follows.

#### 2.1.1. Inclusion Criteria

1. Diagnosed with stroke by magnetic resonance imaging (MRI) or computed tomography (CT) examination for the first time
2. Less than 6 months after onset and no spasticity
3. 25 to 80 years of age
4. Able to understand the physician's orders

#### 2.1.2. Exclusion Criteria

1. Aphasia, severe cognitive impairment, and severe depression
2. Unstable condition, including orthostatic hypotension, sepsis, and epilepsy
3. Unilateral spatial neglect or severe visual impairment
4. Ashworth of affected shoulder, elbow, or wrist and hand larger than 2
5. Heart, lung, liver, kidney, and other vital organs function decline or failure

All subjects exhibited hemiparesis, which was shown by Fugl-Meyer assessment (FMA) (scores range from 0 to 100, with higher scores representing better function). The subjects recruited by this study had variable types of stroke and large FMA range. This was because the original idea of this study was to analyze ERD topography of stroke patients in different conditions and compare the classification accuracy using different channel-selection strategies in order to show that an individualized channel-selection strategy in BCI rehabilitation may be needed. Detailed information of the patients (S1–S15) is shown in Table 1. They gave written consent prior to participating in the study. This study was conducted according to the principles expressed in the Declaration of Helsinki and approved by the Institutional Ethical Committee.

### 2.2. Experimental Paradigm

**Figure 1:**
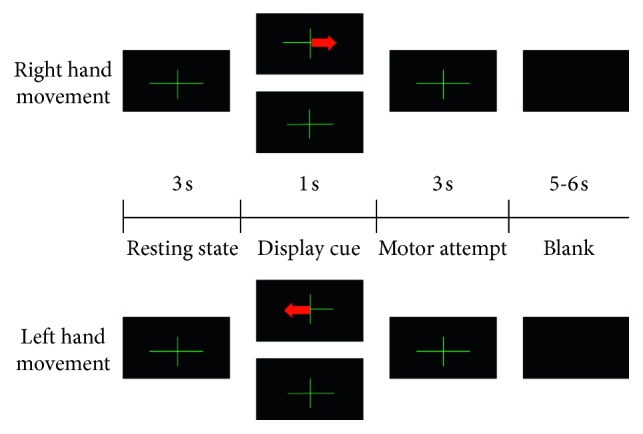
Each trial started with a fixation cross displayed in the center of the screen, which lasted for the whole trial from the first to seventh second. The cue for motor attempt appeared from the fourth to fifth second as a red arrow pointing to the right or left side specifying the task to be performed. When no arrow appeared, it was the blank control, during which the subject need not do anything but stare at the cross center. Patients were required to stop the task when the cue disappeared at the end of the seventh second, and only the fixation cross was shown till the end of the trial. To avoid adaptation to the timing, cues were presented in randomized sequence with randomized intervals between 5 seconds and 6 seconds between each trial.

The experiment was conducted between patients and the experimenter in a quiet dark room. Each patient was seated in a comfortable chair with arms supported, and the experimenter explained the task to each patient beforehand. A cue-based paradigm was applied using OpenViBE V2.0.1. A 15-inch computer monitor was placed about 1 meter in front of the subjects. Each patient was requested to stare at the center of the monitor and respond to the cue. Two sessions were conducted for each patient, and each run consisted 40 trials. As shown in Figure 1, each trial started with a fixation cross for 3 seconds, at which the patient was required to stare to avoid excessive eye movements. After this, an arrow cue appeared on the monitor when the motor attempt task needed to be performed. Motor attempt has been proved to have better performance than motor imagery in BCI [21]. In total, 20 arrow cues and 20 blank controls were demonstrated in each session in a randomized order. In the first session, the patient was asked to attempt opening the palm of the paretic hand when the arrow pointed to the paretic side even though the movement of the paretic hand could not be truly executed; when the arrow did not appear (blank control), the patient need not do anything but stare at the cross center. The first session was used to classify between the motor intention of the paretic hand and the resting state, which was needed in BCI therapy, rather than to classify motor intention of the affected hand and the unaffected hand. So, in the second session, ERD topography during movement of the unaffected hand was also collected only for comparison. In the second session, the patient was asked to attempt opening the palm of the unaffected hand when the arrow pointed to the unaffected side; when the arrow did not appear (blank control), the patient need not do anything but stare at the cross center. To avoid adaptation to the timing [22], cues were presented in randomized sequence with randomized intervals between 5 seconds and 6 seconds between each trial. Each trial lasted 7 seconds, and each session lasted about 9 minutes. A 5 minutes' rest was given after the first session to avoid fatigue.

### 2.3. EEG Recordings and Data Processing

During the experiments, EEG data were recorded with ANT eego™rt from 64 Ag/AgCl electrodes, positioned according to the international 10/20 system and streamed through OpenViBE V2.0.1, with CPz as reference and AFz as ground, digitally sampled at 500 Hz with 24-bit resolution. Electrode impedances were kept below 5 KΩ. EEG recordings from all 64 channels were raw data without any preprocessing by the acquisition software.

The EEG data were preprocessed in EEGLAB 14.1.2b (EEGLAB toolbox, Swartz Center for Computational Neurosciences, La Jolla, CA; https://sccn.ucsd.edu/eeglab). The 32nd channel EOG was excluded from the EEG data. The raw data were referenced using an average reference [8, 23]. Then, the data were filtered using a finite impulse response (FIR) bandpass filter (1–40 Hz), and the baseline was removed.

The preprocessed EEG data in each session were divided into 40 trials based on the trial marks, with each epoch containing 220500 data points for all 63 channels. 40 epochs of data were categorized into two groups: (i) motor attempt group and (ii) blank control group. Each trial was detected one by one. If artefacts were visually detected in the trial, then the trial was processed using independent component analysis (ICA) in EEGLAB 14.1.2b using runica ICA algorithm [24]. If the artefacts can still be observed, the corresponding trial was excluded from the following analysis. Time-frequency analysis of each epoch was conducted using Morlet Wavelet [25] in alpha (8–13 Hz) and beta (13–30 Hz) range, respectively, with a step of 0.5 Hz. Then, power spectral density (PSD) of alpha and beta band for all channels in the same group was averaged across epochs to maximize features and minimize noises. Based on the ERD calculation [26], modified ERD ratio was calculated: for each channel, integration across time and frequency in each frequency band was calculated in resting state and moving state, respectively. The two states were separated by the event marks from the cues. With the results of the integration of both states, the ERD ratio was calculated using the following equation in order to quantify the changes of the spectral power in a certain frequency range at a channel  *c*:

(1) $$\begin{array}{c} \text{ERD }{\text{ratio}}_{c}=\frac{E_{1}-S_{1}}{E_{2}-S_{2}}\cdot \frac{\sum_{f=l}^{h}\sum_{t=S_{2}}^{E_{2}}1/R\cdot {\text{spe}}_{c}}{\sum_{f=l}^{h}\sum_{t=S_{1}}^{E_{1}}1/R\cdot {\text{spe}}_{c}}, \end{array}$$

where frequency ranges from *l* to *h*(Hz), sampling rate is *R*(Hz), *S* and *E* are the start and end time points of each state: 1 indicates the resting state and 2 indicates the moving state, spe_*c*_(*μ*V^2^/Hz · ms) is the power spectral density.

The ERD/ERS are not phase locked to the event, and ERD/ERS are highly frequency band-specific signals [27]. Therefore, in our study, the frequency band with lower ERD ratio was selected to represent motor intention. Then, the corresponding calculated ERD ratio of each channel for each subject was plotted as ratio maps.

### 2.4. Classification Accuracy

The accuracy of classifying between motor attempt of the paretic hand and the resting state was calculated using MATLAB (MathWorks Inc., USA). In this paper, we applied three strategies to select the EEG channels for analyzing the classification performance. The first strategy, SM1-4, was to select 4 channels on the contralateral SM1, either Cz, C1, C3, C5, or Cz, C2, C4, C6, according to the side of the affected hand [28]. The second strategy (SM1-5) was similar with the first one which selected 5 channels on the contralateral side, either C1, C3, C5, FC3, CP3, or C2, C4, C6, FC4, CP4 [26]. It was believed these two classical channel-selection practices could close the loop between the original brain region of motor control and the actual movement. The third strategy, ERD selection, was to select 4 channels with the lowest ERD ratio to formulate the individualized loop of motor intention and motor attempt according to each patient's condition. The selected 40 trials of EEG data were divided into two groups, namely, the motor attempt of the paretic hand and resting state. In addition to the data processing mentioned above, spatial filtering was also conducted using the common spatial pattern (CSP) in order to extract features for classification [29]. The dimension of the feature matrix is two times as many as numbers of the selected channels. After the feature extraction, the selected features were then classified in a classifier based on support vector machine (SVM) algorithm. The linear kernel was applied [30, 31]. The accuracy of a 5-fold cross-validation test, using 80% data sets for training and 20% for testing, was calculated. ANOVA with repeated measures was applied for statistical analysis to analyze the difference in the classification accuracy between the channel-selection strategies.

## 3. Results

### 3.1. ERD Topography

**Figure 2:**
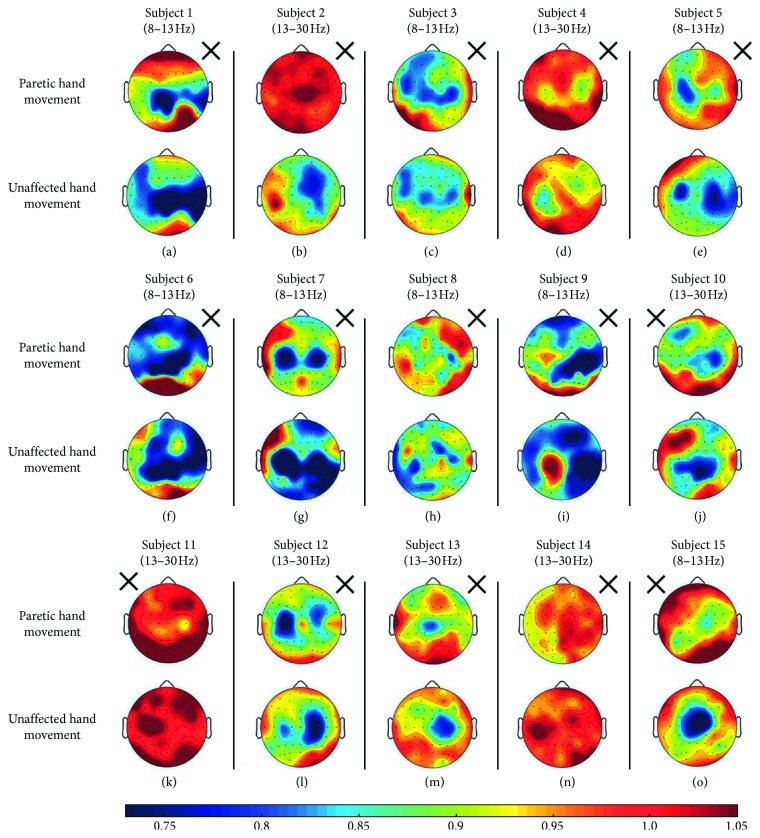
ERD ratio-maps: ERD activation during paretic hand movement and unaffected hand movement relative to resting state overlaid on topography, respectively, for 15 stroke patients. The cross indicates the side of the paretic hand. Blue regions indicate the involved areas when ERD occurs during mental tasks.

**Figure 3:**
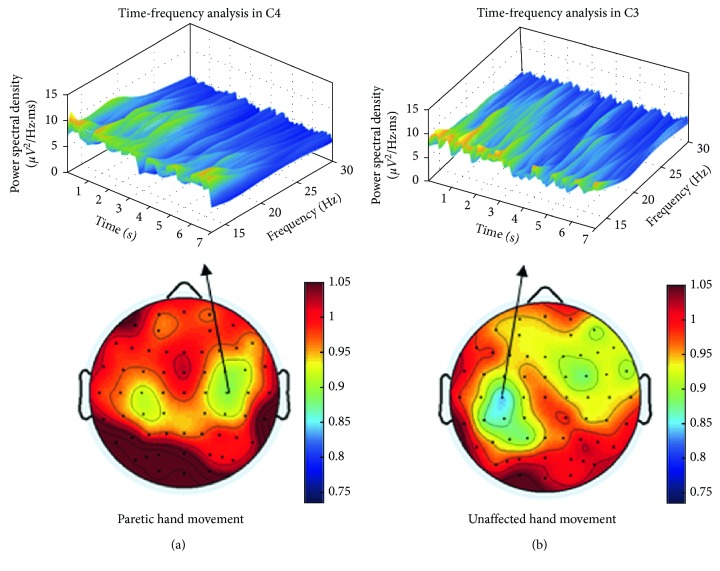
Time-frequency graph and ERD topography during motor attempt for S4. During the motor attempt of the paretic or unaffected hand, based on Morlet Wavelet time-frequency analysis, no apparent decline was found in power spectral density during the experiment. Correspondingly, no ERD was shown in the ERD ratio-maps. (a) Paretic hand movement. (b) Unaffected hand movement.

As shown in Figure 2, during the motor attempt of the unaffected hand, ERD was detected in alpha and/or beta frequency bands in S1, S2, S3, S5, S6, S7, S8, S9, S10, S12, S13, and S15. ERD was not detected in S4, S11, and S14, who were regarded as ERD blind [32]. The example of S4 is shown in Figure 3, in which no apparent decline was found in power spectral density during the experiment. For S1, S2, S3, S5, S6, S7, S9, S12, and S13, ERD was found around C3 and/or C4; while for S8, ERD focused on temporal areas C4 and FC4; for S10, ERD focused on the central parietal area; for S15, ERD focused on the central and frontal central areas.

More patterns of ERD topography were identified during the motor attempt of the paretic hand (Figure 2). The subjects were divided into 5 groups based on the following criteria:

- Whether ERD appears during hand movement or not
- Whether ERD appears on the ipsilateral or the contralateral side
- Whether ERD is identified on specific sites or in a wide range

**Figure 4:**
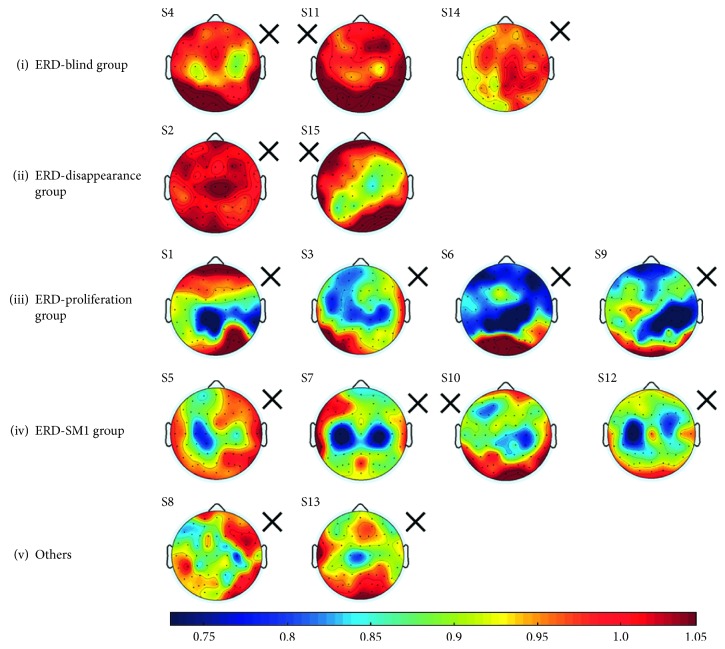
Grouping of the patients: ERD ratio-maps during paretic hand movement relative to resting state. The cross indicated the side of the paretic hand. Blue regions indicate the involved brain areas when ERD occurs during mental tasks.

**Figure 5:**
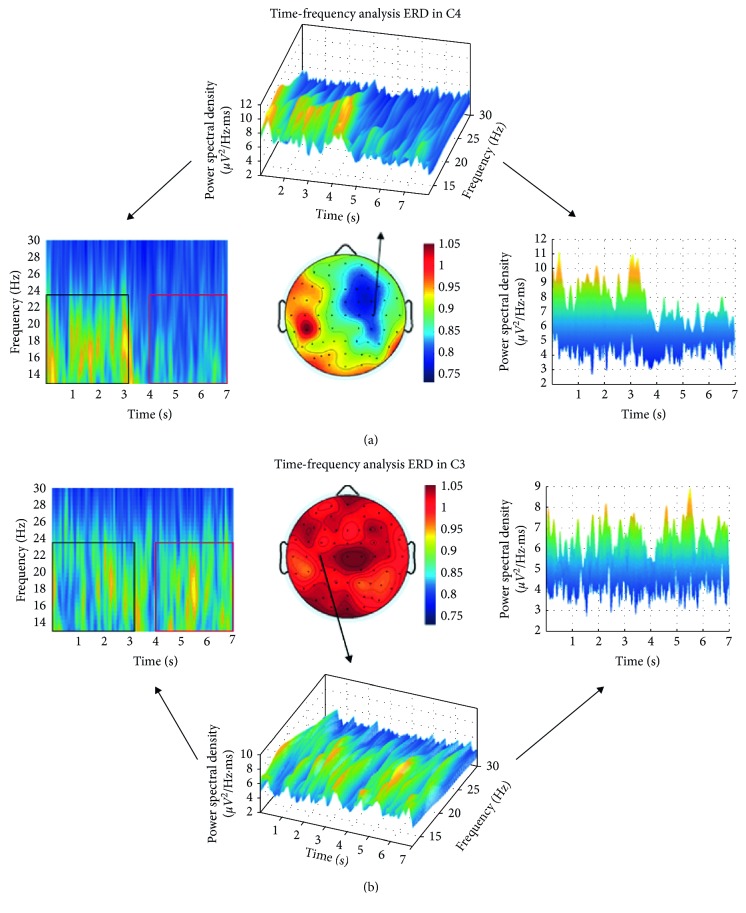
Time-frequency graph and ERD topography during motor attempt for S2. (a) Unaffected band (left) movement: During the first to third second, the subject was in resting state. During the fourth to seventh second, the subject was conducting the required task. During the motor attempt of the unaffected left hand, apparent decline was identified in power spectral density on contralateral C4 compared with the resting state, based on Morlet Wavelet time-frequency analysis. (b) Affected band (right) movement: During the first to third second, the subject was in resting state. From the fourth to seventh second, the subject was required to conduct the motor attempt task. During the motor attempt of the paretic right hand, no apparent decline was found in power spectral density on contralateral C3 compared with resting state, based on Morlet Wavelet time-frequency analysis. Correspondingly, no ERD was shown in the ERD ratio-map.

Specific grouping of the patients is shown in Figure 4 and listed as follows:

- ERD-blind group: for S4, S11, and S14, no ERD was found in alpha and/or beta frequency bands over the affected and/or the unaffected hemispheres.
- ERD-disappearance group: S2 and S15 were not ERD blind as ERD was identified during their movement of the unaffected hand. So the disappearance of ERD during the motor attempt of the paretic hand may be due to the deficits in the affected hemisphere. Time-frequency analysis of EEG during the paretic hand motor attempt and unaffected hand motor attempt for S2 is shown in Figure 5.
- ERD-proliferation group: for S1, S3, S6, and S9, ERD was detected in a wide range of electrodes covering brain regions with a stronger focus on the unaffected hemispheres. For S3, ERD was found on the bilateral hemispheres with a stronger focus on the sensorimotor cortex area.
- ERD-SM1 group: for S5, S10, and S12, ERD focused on the C3 or C4 of the affected hemisphere, whereas for S7, ERD focused on the bilateral C3 and C4.
- Others: for S8, slight ERD was detected in C4. For S13, slight ERD was detected in C1 and Cz.

### 3.2. Channel-Selection Strategies and Classification Performance

The accuracy of classifying motor attempt of the paretic hand and resting state is shown in Table 2, applying different channel-selection strategies. Since no ERD was detected for S2, S4, S11, S14, and S15 during the motor attempt of the paretic hand, the classification accuracy was not calculated. The classification accuracy for all other subjects was above chance-level (50%). The classification accuracy using ERD selection was at least 7.5% higher than that using SM1-4 for S1, S3, S5, S6, and S9. For S7, S8, S10, S12, and S13, the results yielded by SM1-4 was 2.5% or 5% lower than that by ERD selection. The classification accuracy using ERD selection was at least 10% higher than that using SM1-5 for S1, S3, and S10. The accuracy generated by SM1-5 was 2.5% or 5% lower than that by ERD selection for S6, S7, S8, S9, S12, and S13. Results were equal for S5. For the ERD-proliferation group, the accuracy of ERD selection strategy was 79.38 ± 10.28%, higher than that of SM1-4 (70.00 ± 9.35%) or SM1-5 (71.25 ± 8.54%) selection strategy. For the ERD-SM1 group, the accuracy among these three channel-selection strategies was slightly different (SM1-4 selection: 67.50 ± 3.54%, SM1-5: 70.00 ± 4.56%, ERD selection: 71.88 ± 3.75%). One-way ANOVA with repeated measure determined that mean classification accuracy differed significantly between different channel-selection strategies (*F* (2, 18) = 19.442, *p* < 0.0005). Post hoc tests using the Bonferroni correction revealed that there was a significant difference in the classification accuracy between the SM1-4 channel-selection strategy (69.50 ± 8.48%) and ERD Selection (75.75 ± 8.98%) (*p*=0.001) and between the SM1-5 channel-selection strategy (71.25 ± 7.84%) and ERD Selection (75.75 ± 8.98%) (*p*=0.015). However, there was no significant difference in the classification accuracy between the SM1-4 channel-selection strategy (69.50 ± 8.48%) and SM1-5 channel-selection strategy (71.25 ± 7.84%) (*p*=0.134). Therefore, we can conclude that ERD channel-selection strategy generated a statistically significant increase in classification accuracy than the other two classical channel-selection strategies.

## 4. Discussion

### 4.1. ERD Topography and Clinical Assessment

The results showed ERD topography varied during the motor attempt of the paretic hand due to the deficits caused by stroke [17]. Based on the pattern of ERD topography and clinical assessment, the patients' condition can be further inferred. (i) ERD-blind group: ERD blind subjects were not taken into consideration. (ii) ERD-disappearance group: ERD was found during movement of the unaffected hand but not found during the motor attempt of the paretic hand. It can be inferred that the expression of motor intention of the paretic hand may be influenced by the lesion, which can also be demonstrated by a rather low FMA. (iii) ERD-proliferation group: For S6 and S9, ERD was shown in a wide range of electrodes covering brain regions with areas compensating for the expressions of motor intention by SM1 in the affected hemisphere. While for S1 with higher FMA than that of S6 and S9, there was a stronger focus of ERD around CP1, CPz, and TP8. For S3 who was also evaluated with a rather high FMA, ERD was found on bilateral hemispheres with a stronger focus on SM1. It can be inferred for S3, recruitment of compensatory brain regions may narrow down and ultimately activation area of motor control may focus on the contralateral SM1 again [20]. (iv) ERD-SM1 group: For S7, S10, and S12, ERD was found around SM1 in the affected hemisphere, which means SM1 was still capable of expressing motor intention. According to the fact that these 3 subjects had high FMA, we can infer that SM1 was preserved or has recovered from the brain lesion. However, for S5, the day of experiment was only 8 days after stroke onset. Although motor intention was detected on SM1, the neural pathway between cortical activity and actual movement could be seriously affected, which may be the cause of a low FMA. (v) Others: S13 was diagnosed with hemorrhage in the left basal ganglia, which may have a serious effect on motor function, leading to a rather low FMA. The electrode-detecting motor intention shifted to a narrow area as shown in the ERD topography. Although the electrode-detecting motor intention shifted to C4 on the unaffected hemisphere for S8, the infarction may have less severe effects on motor control as demonstrated by a higher FMA than the hemorrhage for S13 [33]. Brain reorganization is influenced by numerous factors, such as lesion site, lesion type, and TFO. ERD topography can be used as an additional supplement for clinical evaluation for stroke patients' condition, especially the progress of brain reorganization, as it can indicate the brain regions expressing motor intention during motor attempt of the paretic hand.

### 4.2. Channel-Selection Strategies and Classification Performance

The aim of this study focused on investigating the expression of motor intention, which is an important element of the BCI neural circuit [34]. The offline motor intention classification accuracy showed the performance of the classifier and thus can predict the online BCI classification performance. Thus, the offline motor intention classification accuracy was calculated and taken into researching along with ERD topography and clinical assessment. Table 2 shows the offline classification accuracy generated by different channel-selection strategies. The first classical SM1-4 channel-selection performance was compared with the individualized ERD channel-selection strategy: (i) ERD-blind group and (ii) ERD-disappearance group: patients in the ERD-blind group and ERD-disappearance group were not taken into consideration. (iii) ERD-proliferation group: the classification accuracy using ERD selection was at least 7.5% higher than that using SM1-4. In these subjects, ERD was detected in a wide range of electrodes covering brain regions including the unaffected hemisphere which compensated for the affected motor function. Therefore, more effective classification features can be extracted from the channels showing ERD, which resulted in a much higher classification accuracy applying individualized channel selection strategy. (iv) ERD-SM1 group: For S7, S10, and S12, ERD was shown around C3 and/or C4 of the affected hemisphere. The motor intention of these 3 subjects was mainly expressed on SM1. Consequently, classification accuracy analyzed by the individualized channel selection strategy was similar with that analyzed by classical channel selection strategy. Even so, individualized channel selection can detect the principal channels representing ERD more accurately which results in slightly higher accuracy. However, for S5, ERD was detected limitedly on C3, CP1, FC3, and C1 without a wide range, and the features could be extracted more effectively, which resulted in higher accuracy (7.5% increase) using ERD selection in spite of overlapping 2 channels in affected SM1. (v) Others: ERD was detected in a narrow area consisting of one or two EEG channels, which means the principal feature of motor intention was expressed in a limited area. The motor intention features can be detected by ERD selection but with limited improvement on classification accuracy. Thus, the classification accuracy using ERD selection was 2.5% higher for S8 and 5% higher for S13 than using SM1 channel-selection strategy. The ERD channel-selection strategy was based on the motor intention features which varied from patient to patient, while classical channel-selection strategies were based on the motor control for healthy subjects [13, 14]. Therefore, the comparison result between SM1-5 channel selection strategy and ERD selection was similar except for S5. For S5, SM1-5 and ERD selection generated equal classification accuracy since the analyzed EEG channels overlapped for these two strategies.

The results indicate that motor intention expression is not limited within SM1 for stroke patients. Depending on the severity of the deficits in the affected motor cortex, two main patterns of cortical reorganization have been identified in a longitudinal study; for stroke patients whose affected motor cortex is extensively damaged, persistent recruitment of additional ipsilateral and contralateral brain regions was found during movement of the paretic hand [20]. Therefore, the compensatory region(s) should be taken into consideration for BCI implementation for patients whose affected motor ability has little potential to recover on the original site. The altered pattern of activated brain regions after stroke may have the potential to predict the regions representing motor functions after neural reorganization. Therefore, the loop between the altered activated brain regions and the paretic hand can be stimulated using individualized channel-selection strategy.

On the contrary, for the patients whose affected motor cortex is damaged within limited extent, another pattern of cortical reorganization appears, in which, after initial recruitment of additional ipsilateral and contralateral brain regions, brain activation during movement of the paretic hand gradually develops toward a pattern of activation restricted to the contralateral sensorimotor cortex; however, this trend of focusing does not imply recovery [20]. It means although the original brain region is still capable of representing motor intention, the neural circuit between the cortex and paretic side of the body may be damaged. Therefore, for the patients whose neural reorganization evolves during the whole rehabilitation process, channel selection in EEG-based BCI rehabilitation can be updated as the expression of motor intention focuses to the contralateral sensorimotor cortex. With an individualized channel selection strategy, BCI rehabilitation training can respond more accurately to the patient's motor intention in order to stimulate the neural circuit, which is the principle of BCI-based rehabilitation.

### 4.3. Limitations

The objective of this paper was to investigate ERD topography of stroke patients in different conditions, and compare the classification accuracy of the motor attempt of the paretic hand using EEG data from SM1 or from the channels showing ERD. However, no definite relationship between lesion type and ERD expression can be concluded. As mentioned above, in this study, a heterogeneous group of patients was recruited with the level of severity ranging from moderate to very severe and with different lesion sites. It is difficult to find a correlation between the patient's ERD topography and FMA, since FMA varied from subject to subject even in the same group due to various factors, including TFO, lesion site, lesion type, etc. In further research, more patients with the same lesion type should be selected, and the quantitative correlation between the ERD topography and these influencing factors can be investigated. Furthermore, the actual rehabilitation effect using EEG-based BCI with the proposed individualized channel-selection strategy also needs to be validated in the future.

## 5. Conclusion

This study analyzed ERD topography in stroke patients during the motor attempt of the paretic hand and compared the classification performance using different channel-selection strategies collecting EEG from different channels. Results showed that the classification accuracy analyzing the channels showing ERD is higher than that analyzing EEG from SM1, which, in addition, may not be able to recover the original motor control ability in stroke patients with severe damages. We believe the findings can explain the reason why the accuracy of classification is rather low for some stroke patients so that they cannot be recruited in the BCI training. The main hypothesis behind stroke rehabilitation with BCI is that closing the loop between motor intention and actual movement can restore functional corticospinal and corticomuscular connections. The results in this study indicated that the closed loop should be individualized according to patient's deficits and condition to achieve a better rehabilitation outcome. The actual rehabilitation effects of larger amount of patients using EEG-based BCI with this channel-selection strategy need to be validated in the future.

## Figures and Tables

**Table 1:** Patient information and clinical evaluation.

Subject	Age	Lesion site	Lesion type	Paretic side	TFO (days)	FMA
S1	39	Left basal ganglia region	Hemorrhage	Right	18	60
S2	66	Left basal ganglia region	Infarction	Right	20	25
S3	32	Left pons	Infarction	Right	36	87
S4	58	Left pons	Infarction	Right	28	35
S5	47	Left basal ganglia region	Hemorrhage	Right	8	10
S6	78	Left pons	Infarction	Right	53	21
S7	62	Left middle cerebral artery area	Infarction	Right	129	49
S8	64	Brainstem	Infarction	Right	13	88
S9	49	Left basal ganglia region Middle cerebral artery area	Hemorrhage	Right	73	45
S10	73	Right basal ganglia region	Infarction	Left	51	56
S11	66	Bilateral basal ganglia regions Parietal lobe	Infarction	Left	46	79
S12	61	Left occipital-parietal lobe	Hemorrhage	Right	72	93
S13	43	Left basal ganglia region	Hemorrhage	Right	170	22
S14	60	Bilateral frontal-temporo-parietal lobe	Infarction	Right	82	24
S15	30	Right temporo-parietal lobe	Hemorrhage	Left	50	45
Average ± SD					57 ± 45	49 ± 27

TFO, time from onset; FMA, Fugl-Meyer assessment.

**Table 2:** Channel-selection strategies and classification performance.

Group	Subject	SM1-4 selection	Accuracy	SM1-5 selection	Accuracy	ERD selection	Accuracy
ERD-blind group	4	None	None	None	None	None	None
	11	None	None	None	None	None	None
	14	None	None	None	None	None	None
Average ± SD			None		None		None
ERD-disappea-rance group	2	None	None	None	None	None	None
	15	None	None	None	None	None	None
Average ± SD			None		None		None
ERD-proliferation group	1	Cz C1 C3 C5	82.5%	C1 C3 C5 FC3 CP3	82.5%	CP1 Pz C1 TP8	92.5%
	3	Cz C1 C3 C5	67.5%	C1 C3 C5 FC3 CP3	67.5%	FC5 C4 C5 C1	80.0%
	6	Cz C1 C3 C5	60.0%	C1 C3 C5 FC3 CP3	62.5%	C4 CP2 AF8 C2	67.5%
	9	Cz C1 C3 C5	70.0%	C1 C3 C5 FC3 CP3	72.5%	C4 CP2 CP6 CP4	77.5%
Average ± SD			70.00 ± 9.35%		71.25 ± 8.54%		79.38 ± 10.28%
ERD-SM1 group	5	Cz C1 C3 C5	67.5%	C1 C3 C5 FC3 CP3	75.0%	C3 CP1 FC3 C1	75.0%
	7	Cz C1 C3 C5	72.5%	C1 C3 C5 FC3 CP3	72.5%	C1 C2 C3 C4	75.0%
	10	Cz C2 C4 C6	65.0%	C2 C4 C6 FC4 CP4	67.5%	C4 AF3 F5 CP4	70.0%
	12	Cz C1 C3 C5	65.0%	C1 C3 C5 FC3 CP3	65.0%	C3 FC3 C1 CP3	67.5%
Average ± SD			67.50 ± 3.54%		70.00 ± 4.56%		71.88 ± 3.75%
Others	8	Cz C1 C3 C5	85.0%	C1 C3 C5 FC3 CP3	85.0%	F7 FC2 C4 F5	87.5%
	13	Cz C1 C3 C5	60.0%	C1 C3 C5 FC3 CP3	62.5%	Cz CP1 C1 C2	65.0%
Average ± SD			72.50 ± 17.68%		73.75 ± 15.91%		76.25 ± 15.91%
Overall average ± SD			69.50 ± 8.48%		71.25 ± 7.84%		75.75 ± 8.98%

SM1-4 selection, 4 channels selected from the sensorimotor cortex. SM1-5 selection, 5 channels selected from the sensorimotor cortex. ERD selection, 4 channels selected with the lowest ERD ratio. SD, standard deviation.

## Data Availability

The EEG data used to support the findings of this study are available from the corresponding author upon request.
